# Design and Experimental Evaluation of a Robust Position Controller for an Electrohydrostatic Actuator Using Adaptive Antiwindup Sliding Mode Scheme

**DOI:** 10.1155/2013/590708

**Published:** 2013-07-22

**Authors:** Ji Min Lee, Sung Hwan Park, Jong Shik Kim

**Affiliations:** School of Mechanical Engineering, Pusan National University, Jangjeon-dong, Geumjeong-gu, Busan 609-732, Republic of Korea

## Abstract

A robust control scheme is proposed for the position control of the electrohydrostatic actuator (EHA) when considering hardware saturation, load disturbance, and lumped system uncertainties and nonlinearities. To reduce overshoot due to a saturation of electric motor and to realize robustness against load disturbance and lumped system uncertainties such as varying parameters and modeling error, this paper proposes an adaptive antiwindup PID sliding mode scheme as a robust position controller for the EHA system. An optimal PID controller and an optimal anti-windup PID controller are also designed to compare control performance. An EHA prototype is developed, carrying out system modeling and parameter identification in designing the position controller. The simply identified linear model serves as the basis for the design of the position controllers, while the robustness of the control systems is compared by experiments. The adaptive anti-windup PID sliding mode controller has been found to have the desired performance and become robust against hardware saturation, load disturbance, and lumped system uncertainties and nonlinearities.

## 1. Introduction

Recently, lots of hydraulic servo systems have been replaced by electric motor-driven systems to overcome the drawbacks of traditional hydraulic servo systems such as oil leakage, maintenance, and laying complex pipe. In spite of these drawbacks, hydraulic systems are still used in fields that require large force and high power. In order to solve these issues, electrohydrostatic actuator (EHA) systems are in the process of being developed for industrial use. The EHA systems can also be referred to as a direct drive volume controlled (DDVC) actuator [1–3] or valveless hydraulic servo (VHS) actuator [4].

The EHA systems generally consist of an electric motor, hydraulic pump, hydraulic cylinder, accumulator, check valves, and relief valves for safety. An electric motor that is directly tied to the hydraulic pump controls the cylinder, whose directional change depends upon the electric motor's rotation direction. Also, an electric motor installed in the EHA system controls the flow rate and pressure of the working fluid by regulating the velocity and torque of the motor.

The EHA systems are kind of compact high-power actuator modules that integrate the electric motor, hydraulic pump, hydraulic cylinder, accumulator, check valves, and relief valves into one component by using the manifold block. Compared to conventional hydraulic systems, EHAs are very small and do not need complex pipe lines. The EHA systems are seen as environment-friendly hydraulic servo system because it does not leak oil.

The main advantage of using EHA systems on the whole comes from its increased energy efficiency [5–10]. The EHA systems designed in this research do not use any control valves such as a servo valve and can also supply the minimum energy needed for the hydraulics system by controlling the servo motor, meaning that the EHA systems are extremely energy efficient. Also, by using an electric servo motor as a control device, EHA systems can quickly achieve fast response and high accuracy. However, since the EHA systems are a kind of hydraulic servo system, it has nonlinearity related to the friction force mainly located in the hydraulic cylinder. The EHA system also has the closed hydraulic circuit which can use relatively little working fluid. The performance of the EHA system can also be sensitive to variation within the system parameters of the working fluid such as variety of effective bulk modulus and the leakage coefficient due to changes in the working environment [5, 7, 9, 11–13]. Those nonlinearity and uncertainty may cause the position control system of the EHA to be unstable or have a much degraded performance.

In order to obtain the desirable performance of an EHA position control system, a number of control strategies have been applied and investigated by many researchers. Some of the work has used linear control theory for the position control of EHA [14–16]. The nonlinear robust control schemes such as sliding mode control, back-stepping control, and Fuzzy logic control have been proposed to achieve robustness of position control system for EHA by compensating system nonlinearity and uncertainties and to compensate for the nonlinear dynamics of EHA system such as friction [3, 5, 6, 9–11, 14, 17, 18]. Adaptive control approaches have been also proposed to make up for parameter disturbances such as varying the viscous friction coefficient and the effective bulk modulus due to change of working condition [1–3, 9, 11, 19, 20]. 

However, a fast response with small overshoot should be the objective for the practical use of the EHA position control systems in industrial machine such as plastic injection molding machine, aircraft actuation system, mobile machinery, CNC pipe bending machines, and hydraulic servo control of steering system for ship and hydraulic press [1, 2] even though there are lots of uncertainties and nonlinearity in position control system of EHA. The problem concerning large overshoot from the saturation in the electric motor has been considered in recent studies [2]. In [2], the saturation of voltage and current of the PWM driver and DC power is taken into account for position control system of EHA. The researchers of [2] also consider dead zone torque due to the static friction of motor pump and variable load torque. The PD-PI hybrid control scheme is proposed to control the BLDC motor in order to achieve small overshoot and to compensate for the load disturbances. However, only a computer simulation can verify the proposed linear control scheme although there are lots of uncertainty and nonlinearity besides the dead zone and motor saturation. In [2, 19], they proposed a self-turning Fuzzy PID control scheme to compensate for the dead zone, saturation of motor angular velocity, parameter uncertainties, and load disturbance with experimental verification. However, improved accuracy and fast response appear necessary to apply the position control results to servo press. Also, the design of fuzzy rules depends largely on the experience of experts or input-output data.

In this research, an adaptive antiwindup PID sliding mode control scheme is proposed to solve problems of overshoot in position control caused by saturation of angular velocity to the electric motor. The previous researches considered an antiwindup scheme based on the conventional sliding mode controller [21, 22]. However, the proposed controller consists of an adaptive control and a conventional sliding mode control with antiwindup scheme. Therefore, the proposed control scheme looks to implement robustness to parameter variations in a variety of working condition, nonlinearity, and load disturbance. The validation of the proposed control scheme is verified by experiment.

This paper will proceed as follows: Section 2 reveals the composition and details of the position control test rig of an EHA prototype, followed by system modeling and parameter identification of the EHA systems in Section 3. In Section 4, position controllers for the EHA system using traditional PID, antiwindup PID, and adaptive antiwindup PID sliding mode control schemes are presented. The comparative results of the experimental tests executed on the position control test rig of EHA applying PID, antiwindup PID, and adaptive antiwindup PID sliding mode control schemes are revealed in Section 5, followed by conclusions in Section 6.

## 2. Experimental Setup of the Electrohydraulic Actuator Prototype

In this study, the EHA system consists of a bidirectional brushless direct current (BLDC) motor, axial displacement hydraulic piston pump, acting doubly as a double rod hydraulic cylinder, accumulator, check valves, and relief valves. The accumulator serves as a hydraulic reservoir, pressurizing to obtain improved response speed by increasing the suction ability of the hydraulic pump and to prevent cavitations of the EHA system. The electric motor driving the hydraulic pump directly controls the hydraulic cylinder. The direction change of the hydraulic cylinder depends on the change of rotation direction of the electric motor. Also, an electric motor installed in the EHA system controls the flow rate and pressure of the working fluid by controlling the velocity and torque of the electric motor. The DC power supply, which has a maximum DC power supply capacity of 27 kW, supplies electric power to the electric motor. The detailed specifications of the components are shown in Table 1.

**Figure 1:**
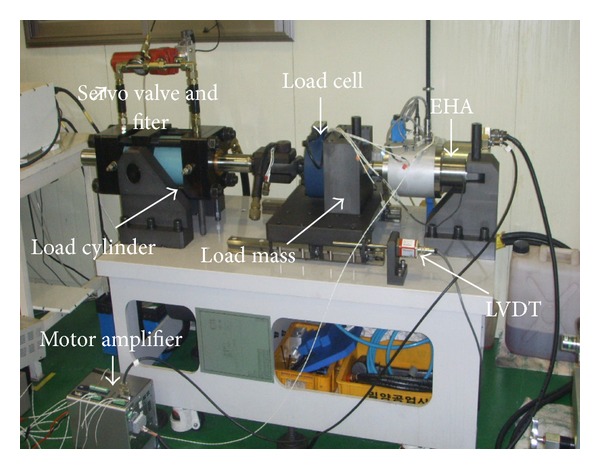
Photo of the position control test rig of the EHA.

**Figure 2:**
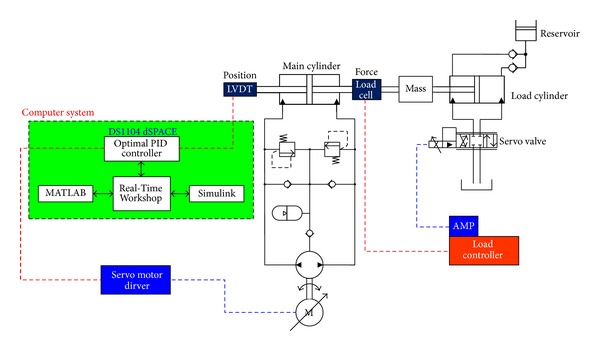
Schematic diagram for the test rig of the EHA prototype.

In this paper, we developed an EHA prototype which can be used for various purposes in industries such as an aircraft actuation system, mobile construction machinery, servo press, steering gear, and injection molding machine, with the overall specifications shown in Table 2.  Figure 1 is a photo of the position control test rig of the EHA system, followed by the schematic diagram of hydraulic position control test rig for the developed EHA prototype in Figure 2.

The test rig for the developed EHA prototype consists of two hydraulic cylinders: a main cylinder which is a part of the EHA and a load cylinder. Two cylinders are directly coupled by a shaft, and the load mass is installed between the two cylinders. The electric motor controls the main cylinder of EHA, while the load cylinder is controlled by the servo valve. 

The hydraulic flow to the main cylinder is controlled by the electric BLDC servo motor operated through a driver directly connected to the proposed controller. The position of the main cylinder gets measured by a linear variable displacement transducer (LVDT) installed on the bracket attached to the load mass, and another one is also installed in the hollow of the main cylinder rod. The signal from the LVDT is used for the closed-loop control of the main cylinder via proposed controllers. The output force gets measured by the load cell installed at the coupling between two cylinders.

DS1104 dSPACE board is used as a main control device to implement proposed position controllers: optimal PID, optimal antiwindup PID controller, and an adaptive antiwindup PID sliding mode controller. The real-time control system is programmed by MATLAB/Simulink and gets transferred to the dSPACE board through the Real-Time Workshop. The parameters of the controller implemented on the board can be changed and monitored through the Control-Desk software in real time.

## 3. Modeling of Position Control System of EHA

In this section, we derive a mathematical model of EHA systems, identifying system parameters by the signal compression method (SCM) in order to design the position controller of EHA. 

### 3.1. Mathematical Model of EHA System

**Figure 3:**
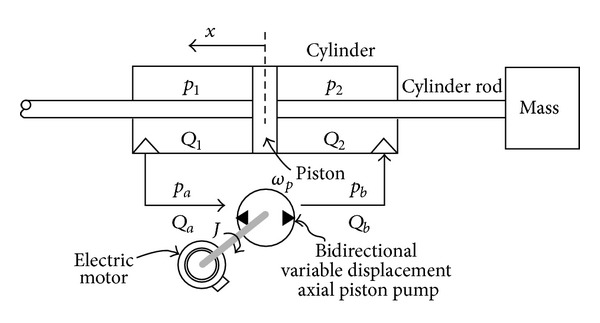
Schematic diagram of an EHA system.

Figure 3 shows the schematic diagram of EHA system. As shown in Figure 3, the pump in/outflows *Q*_*a*_, *Q*_*b*_ can be represented with the pump inlet/outlet chamber pressures *p*_*a*_, *p*_*b*_ and the cylinder displacement *x* as follows:

(1) $$\begin{array}{c} Q_{a}=D_{p}\omega_{p}-\xi \left(p_{a}-p_{b}\right)-\frac{V_{a}}{\beta_{e}}\frac{dp_{a}}{dt}-C_{ep}\left(p_{a}-p_{r}\right),\\ Q_{b}=D_{p}\omega_{p}-\xi \left(p_{a}-p_{b}\right)+\frac{V_{b}}{\beta_{e}}\frac{dp_{b}}{dt}+C_{ep}\left(p_{b}-p_{r}\right), \end{array}$$

where *D*_*p*_ is displacement of the pump, *ξ* is the pump cross port leakage coefficient, *C*_*ep*_ is the pump leakage coefficient, *p*_*r*_ is the accumulator pressure, *β*_*e*_ stands for the effective bulk modulus of the working fluid, and *V*_*a*_, *V*_*b*_ are inlet/outlet volumes of pipe and cylinder chamber, respectively. 

In addition, the cylinder inlet/outlet flows *Q*_1_, *Q*_2_ can be represented by

(2) $$\begin{array}{c} Q_{1}=A\dot{x}+\frac{V_{a}}{\beta_{e}}\frac{dp_{1}}{dt}+L\left(p_{1}-p_{2}\right),\\ Q_{2}=A\dot{x}-\frac{V_{b}}{\beta_{e}}\frac{dp_{2}}{dt}-L\left(p_{2}-p_{1}\right), \end{array}$$

where *A* is pressurized area of hydraulic piston, *L* is internal leakage coefficient of the cylinder, and *p*_1_, *p*_2_ are the cylinder inlet/outlet chamber pressures, respectively. In this study, the external leakage of the cylinder is neglected.

In this study, *V*_*a*_ and *V*_*b*_ are assumed to be equal due to the double rod, and double acting hydraulic cylinder is used in EHA system. Also, *V*_*a*_ and *V*_*b*_ can be expressed with the nominal volume of each EHA chamber *V*_0_ which equals the mean volume of pipe and cylinder chamber as follows:

(3) $$\begin{array}{c} V_{a}=V_{0}+Ax,\\ V_{b}=V_{0}-Ax. \end{array}$$

Since a solid tubing is used in the prototype, the pressure impact of pipe expansion and flexibility on the effective bulk modulus is assumed to be negligible. Therefore, the relationship pump port flows (*Q*_*a*_, *Q*_*b*_) and cylinder chamber flows (*Q*_1_, *Q*_2_) can be expressed as

(4) $$\begin{array}{c} Q_{1}=Q_{a},\\ Q_{2}=Q_{b}. \end{array}$$

Then, the load *Q*_*L*_ can be defined as follows [21]:

(5) $$\begin{array}{c} Q_{L}=\frac{Q_{1}+Q_{2}}{2}=\frac{Q_{a}+Q_{b}}{2}. \end{array}$$

The relationship between the pump port pressures (*p*_*a*_, *p*_*b*_) and cylinder chamber pressures (*p*_1_, *p*_2_) can then be expressed as

(6) $$\begin{array}{c} p_{a}=p_{1}+p_{\text{pipe}},\\ p_{b}=p_{2}-p_{\text{pipe}}, \end{array}$$

where *p*_pipe_ is pressure drop.

Due to the short length of tubing, pump port pressures are assumed to be equal; that is, the pressure drop can be neglected, to the actuator inlet and outlet pressures such that [7, 8, 11, 17, 22]

(7) $$\begin{array}{c} p_{a}=p_{1},\\ p_{b}=p_{2}. \end{array}$$

Since the symmetrical double-rod hydraulic cylinder is used in this EHA system, the following equation for the cylinder chamber pressures can be established:

(8) $$\begin{array}{c} \frac{dp_{1}}{dt}=-\frac{dp_{2}}{dt}. \end{array}$$

By combining (1), (2), (5), (7), and (8), a simplified pump/cylinder model equation can be expressed as

(9) $$\begin{array}{c} D_{p}\omega_{p}=A\dot{x}+\frac{V_{0}}{\beta_{e}}{\dot{p}}_{L}+C_{T}p_{L}+f_{1}, \end{array}$$

where *p*_*L*_ = *p*_1_ − *p*_2_, *C*_*T*_ = *ξ* + *L* + (*C*_*p*_/2) is the equivalent leakage coefficient and *f*_1_ is unmodeled dynamics of hydraulic part of the EHA system.

The actuator force and the displacement of the load can be represented as

(10) $$\begin{array}{c} F=p_{L}A=M\ddot{x}+B\dot{x}+f_{2}, \end{array}$$

where *A* is pressurized area of hydraulic cylinder, *M* is mass of load, *B* is viscous friction coefficient and *f*_2_ is lumped uncertain nonlinearities due to external disturbance, the unmodeled friction forces, and other hard-to-model terms of mechanical part of the EHA system.

By combining (9) and (10) and neglecting *f*_1_ and *f*_2_, the linear transfer function between the angular velocity of the pump and the displacement of the main cylinder can be expressed as

(11) Gh(s)=x(s)ωp(s)=(ADpβeMV0)(s3+(BM+CTDpβeV0)s2          +βe(BCTDp+A2)MV0s)−1,

where *s* is the Laplace operator.

Based on (11), the variation of the effective bulk modulus of the working fluid can increase or decrease the compressibility of the working fluid, and then the natural frequency of the EHA system can be adjusted, meaning that the variation of the system parameters can affect the performance of the EHA position control system.

On the other hand, the dynamics of the BLDC motor with controller, that is, electric part of the EHA system, is considered as [14, 23]

(12) $$\begin{array}{c} G_{m}\left(s\right)=\frac{\omega_{p}\left(s\right)}{v_{r}\left(s\right)}=\frac{K_{m}\left(T_{1}s+1\right)}{T_{m_{1}}s^{2}+T_{m_{2}}s+1}, \end{array}$$

where *K*_*m*_, *T*_1_, *T*_*m*_1__, and *T*_*m*_2__ are parameters of the BLDC motor with controller.

### 3.2. Parameter Identification

In general, the unknown system parameters of the linear elements in a nonlinear system can be identified by the signal compression method (SCM) [24]. In this study, SCM is applied to identify the hydraulic part of the EHA prototype. 

However, hydraulic servo systems engage in significant nonlinear behavior due to system uncertainties such as pipe losses, leakages, and parameter variations of the working fluid [9–11, 16, 17, 19]. Some of those uncertainties are sometimes neglected because the SCM can only identify the linear part of the system in the identification process. This study then compensates for the neglected nonlinear behavior of the EHA system by using a robust controller, that is adaptive PID sliding mode control scheme.

**Figure 4:**
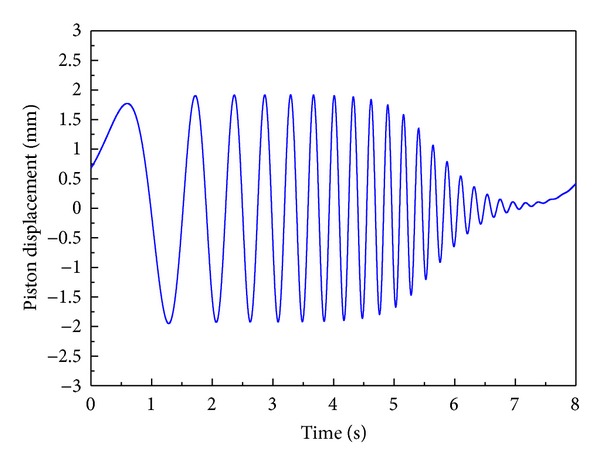
Test signal.

**Figure 5:**
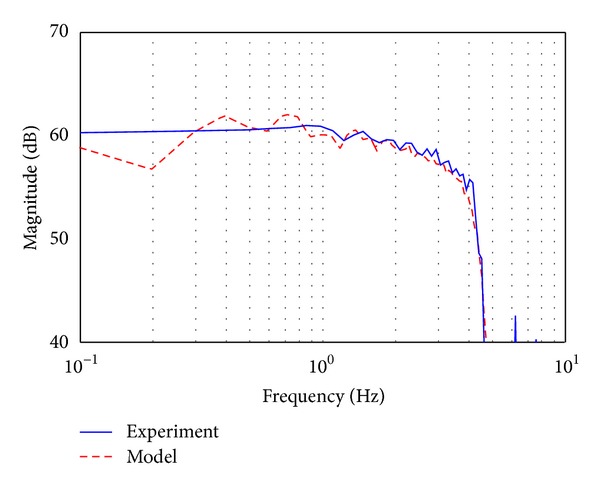
Bode plots of the closed loop nominal model and the closed loop actual system with a proportional controller.

As shown in Figure 4, the test signal that has the same amplitude up to 4 Hz in the frequency domain is applied to the position control system of EHA by constructing a close-loop proportional control system to obtain a more accurate equivalent impulse response for the precise identification of unknown parameters.  Figure 5 shows the comparison of the frequency response of the position control system between the closed-loop nominal model and the closed-loop actual system with a proportional controller, whose gain is 1,000.

The identified transfer function of a closed-loop actual system with a proportional controller for EHA position control system is as follows:

(13) $$\begin{array}{c} \frac{KG\left(s\right)}{1+KG\left(s\right)}=\frac{11.8\times 10^{7}}{s^{3}+2231s^{2}+71.8\times 10^{5}s+11.8\times 10^{7}}, \end{array}$$

where *K* is proportional gain of closed-loop actual system.

From the identified nominal model for the closed-loop position control system for EHA with the proportional controller, the parameters of the position control system can be acquired by eliminating the effect of the proportional controller mathematically.

After eliminating the effect of the proportional controller, the identified transfer function of the hydraulic part of the EHA prototype is as follows:

(14) $$\begin{array}{c} G\left(s\right)=\frac{11.8\times 10^{4}}{s^{3}+2231s^{2}+71.8\times 10^{5}s}. \end{array}$$

**Figure 6:**
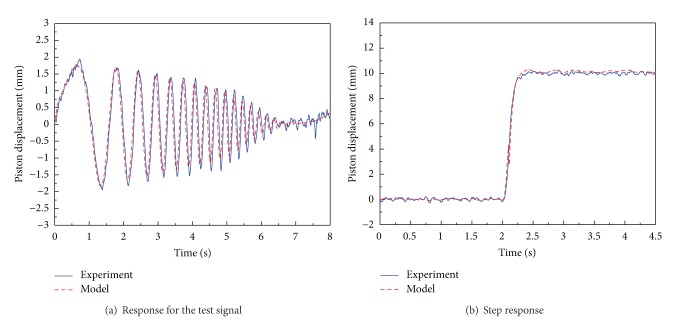
Time responses of the position control system obtained by the experiment and nominal model.

The identified system parameters were verified on the time domain. Figures 6(a) and 6(b) show the response against test signal and the step responses of the nominal model and actual system, respectively. The validations of the identified parameters are estimated quantitatively by the cross-correlation as shown in [24]

(15) $$\begin{array}{c} \text{Corr}=\frac{\sum_{k=1}^{N}\left(Y_{p}\left(k\right)-{\overset{-}{Y}}_{p}\right)\left(Y_{m}\left(k\right)-{\overset{-}{Y}}_{m}\right)}{\sqrt{\sum_{k=1}^{N}\left(Y_{p}\left(k\right)-{\overset{-}{Y}}_{p}\right)}\sqrt{\sum_{k=1}^{N}{\left(Y_{m}\left(k\right)-{\overset{-}{Y}}_{m}\right)}^{2}}}, \end{array}$$

where *Y*_*p*_(*k*) and *Y*_*m*_(*k*) are the responses of the real and nominal model in time train *k*, respectively. ${\bar{Y}}_{p}$ and ${\bar{Y}}_{m}$ are the mean values of *Y*_*p*_(*k*) and *Y*_*m*_(*k*). *N* is the acquired data number. The value of the cross-correlation is 0.904 which can be calculated from the identification results. The identified system parameters are then available to controller design. The identified transfer function in (14) is used to obtain the optimal control gains for designed position controllers by using MATLAB/Simulink.

**Figure 7:**
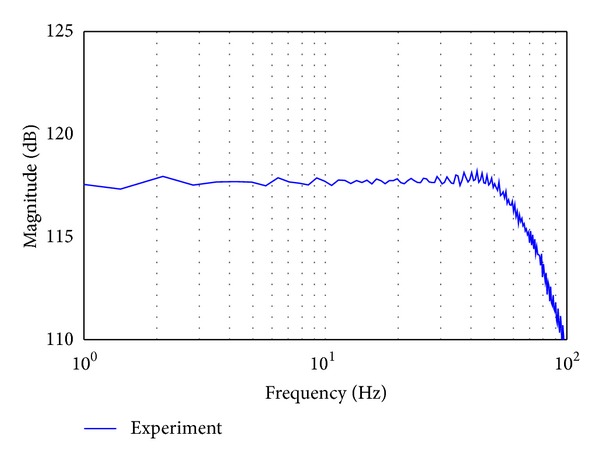
Bode plots of the open-loop actual BLDC motor with controller system.

In addition, the dynamics characteristic of the BLDC motor with controller is also investigated by using SCM. The transfer function of the BLDC motor with controller can be simplified to a second-order linear system as described in (12).  Figure 7 shows bode plot of the actual BLDC motor with controller. The bandwidth of the actual open loop BLDC motor with controller is about 400 rad/s. The bandwidth of the BLDC motor with controller is 20 times larger than that of hydraulic part of the EHA system. Therefore, in this study, we consider that the dynamics of BLDC motor with controller is negligible because the motor dynamic is sufficiently faster than hydraulic part of the EHA. Then, the electric part of the EHA system is taking account of the motor gain *K*_*v*_, and current saturation is reflected in the controller design and the computer simulations stage. 

## 4. Position Controller Design for a Prototype EHA

In this study, an EHA prototype is developed for application in the industrial fields. As mentioned in Section 2, performance characteristics such as no overshoot and rapid response time in position control are the typical demands of the EHA for industrial use. Therefore, appropriate control strategy should be applied to EHA position control system in order to achieve these outcomes.

**Figure 8:**
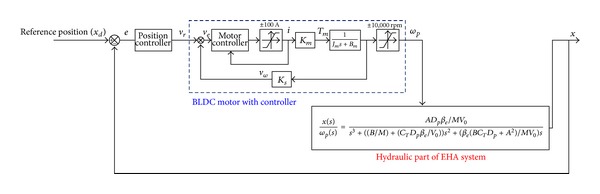
Block diagram for the position control system of the EHA.

Figure 8 shows the block diagram of position control system of the EHA. As mentioned in the previous section, in Figure 8, the dynamics of the BLDC motor with controller is considered as just the motor gain *K*_*m*_, and saturation of current of the BLDC motor is considered as an actuator constraint.

In order to compare and show the desirable performance characteristics for the proposed EHA control system, three kinds of position controllers are designed based on identified simple transfer function. First, an optimal PID controller is designed and applied to the system in order to understand the basic characteristics of EHA position control system. The optimal antiwindup PID controller is then designed to reduce overshoot due to saturation of current in the electric motor. Finally, an adaptive antiwindup PID sliding mode controller is designed to obtain the desirable performance and robustness against the effect of hardware saturation, load disturbance, and parameter variation. 

### 4.1. Optimal PID and Optimal Antiwindup PID Control Systems

An optimal PID position control system is designed to understand basic control performance of EHA prototype. Also, an optimal antiwindup PID control system for the EHA is designed to obtain desirable performance characteristic considering saturation in electric motor. 

The cost function is considered during the design of the optimal PID and optimal antiwindup PID controllers as follows [25]:

(16) $$\begin{array}{c} J\left(K_{p},K_{i},K_{d}\right)=\sum_{t=0}^{\infty }\left(y_{\text{step}}\left(t\right)-y_{\text{step}}^{d}\left(t\right)\right), \end{array}$$

where *y*_step_^*d*^(*t*) is the desired step response of the optimal PID and optimal antiwindup PID control systems and *y*_step_(*t*) is the step response by the identified transfer function in (14). The optimal PID and optimal antiwindup PID controller designs can be stated as

(17) $$\begin{array}{c} \underset{K_{p},K_{i},K_{d}}{\min}J\left(K_{p},K_{i},K_{d}\right), \end{array}$$

where *K*_*p*_, *K*_*i*_, and *K*_*d*_ are the proportional, integral, and differential control gains, respectively.

There are many optimization algorithms in the optimization toolbox of MATLAB/Simulink. The cost function is given by (*K*_*p*_, *K*_*i*_, *K*_*d*_). And the gains of the optimal PID and optimal antiwindup PID controllers *K*_*p*_, *K*_*i*_ and *K*_*d*_ can be found by using the optimization toolbox of MATLAB/Simulink. To find optimal control gains, the reference step input is applied in optimization process, and the response performance is set as follows. The amplitude of reference is 20 mm, the rising time is 0.4 seconds, settling time is 0.8 seconds, percent overshoot is under 5%, and steady-state error is under 0.1 mm.

In the optimization process of optimal PID and optimal antiwindup PID controllers, system parameter uncertainties due to the modeling error are considered. Specifically, ±10% of modeling error is considered in terms of identified transfer function associate with variable system parameters such as effective bulk modulus of the working fluid and total leakage coefficient. 

**Figure 9:**
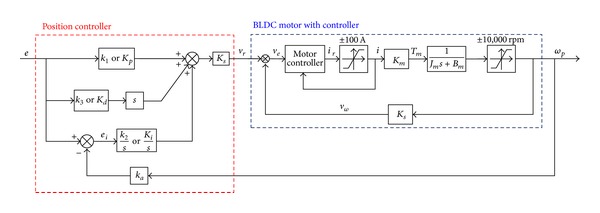
Block diagram for adopted antiwindup algorithm in controller design.

With respect to optimal antiwindup PID and antiwindup PID sliding mode controllers, the antiwindup algorithm shown in Figure 9 is used to consider saturation in an electric motor [2, 26–28]. 

**Figure 10:**
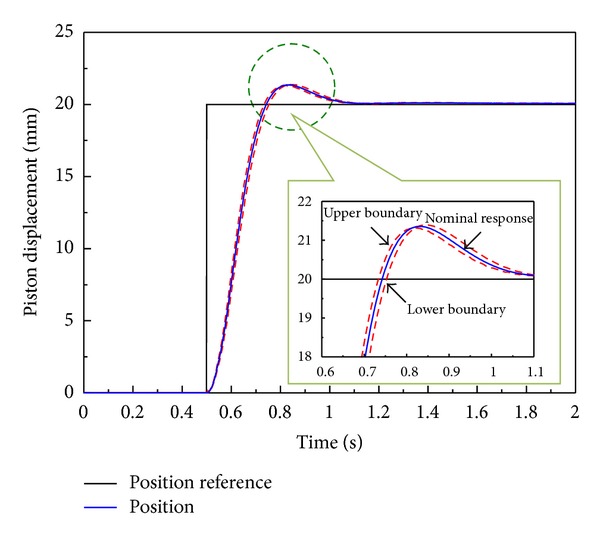
Step response of the optimal antiwindup PID control system in computer simulation based on identified transfer function of EHA system.

Figure 10 shows the results of a computer simulation with optimal antiwindup PID control gains using MATLAB/Simulink optimization toolbox based on identified transfer function considering model uncertainty. The dotted line there shows upper bound and lower bound considering the ±10% of modeling error, and straight line shows nominal simulation result with optimal antiwindup PID control gains. As shown in Figure 10, the simulation result with optimal gains satisfies set response performance.

### 4.2. Adaptive Antiwindup Sliding Mode Controller Considering the PID Sliding Surface

In this study, the adaptive antiwindup PID sliding mode control scheme is also designed to achieve desirable performance and robustness of EHA position control systems. Throughout the controller design process, the effective bulk modulus and leakage coefficients are considered as variable system parameters. In order to consider saturation in electric motor, the antiwindup algorithm presented in Figure 8 also applies to the adaptive antiwindup PID sliding mode control scheme.

The PID sliding surface *ζ* for the design of the adaptive sliding mode control system is defined as [29–32]

(18) $$\begin{array}{c} \zeta =k_{1}e+k_{2}\int e\;dt+k_{3}\dot{e}, \end{array}$$

where the tracking error of the position *e* = *x*_*d*_ − *x*, *x* is the displacement of hydraulic cylinder and *k*_1_, *k*_2_, and *k*_3_ are positive design parameters. 

The sliding mode control law consists of equivalent and robust control term; that is,

(19) $$\begin{array}{c} \omega_{p}=\omega_{p,eq}+\omega_{p,\text{robust}}. \end{array}$$

By combining (11) and (18) with consideration of the noise term in the jerk $\dddot{x}={\dddot{x}}_{m}+{\dddot{x}}_{n}$, the derivative of the sliding surface $\dot{\zeta }$ can be written as

(20) ζ˙=−k3ADpβeBV0+CTDpMβeωp+k3MV0BV0+CTDpMβe(x⃛m+x⃛n)+k3(A2+BCTDp)βeBV0+CTDpMβex˙+k3x¨d+k1e˙+k2e+k3BV0+CTDpMβef,

where $f=f_{1}+\left(C_{T}/A\right)f_{2}+\left(V_{0}/AD_{p}\beta_{e}\right){\dot{f}}_{2}$ is lumped uncertain nonlinearities of the EHA system and the subscripts *m* and *n* denote the nominal and noisy values, respectively.

To determine the equivalent control term *ω*_*p*,*eq*_, the noise term in the jerk is neglected, and it is assumed that the sliding surface *ζ* is at steady-state, that is, $\dot{\zeta }=0$, and then the equivalent control law can be determined as

(21) ωp,eq=BV0+CTDpMβeADpβe×{MV0BV0+CTDpMβex⃛m+(A2+BCTDp)βeBV0+CTDpMβex˙  +k1k3e˙+k2k3e+x¨d}.

Thus, $\dot{\zeta }$ can be rewritten as

(22) $$\begin{array}{c} \dot{\zeta }=k_{3}\frac{MV_{0}}{BV_{0}+C_{T}D_{p}M\beta_{e}}{\dddot{x}}_{n}+\frac{k_{3}}{BV_{0}+C_{T}D_{p}M\beta_{e}}f. \end{array}$$

In the standard sliding mode control, to satisfy the reachability condition that directs system trajectories toward a sliding surface where they remain and to attenuate chattering in the control input, the derivative of the sliding surface is selected as [33, 34]

(23) $$\begin{array}{c} \dot{\zeta }=-D\zeta -K\mathrm{sgn}\left(\zeta \right), \end{array}$$

where *D* and *K* are positive design parameters. 

To determine a robust control term *ω*_*p*,robust_, that achieves robustness to uncertainties such as noise terms in the jerk and unmodeled system dynamics as well as the disturbance force, it is assumed that

(24) $$\begin{array}{c} D_{0}\left|\zeta \right|+K_{0}>\left|\frac{k_{3}MV_{0}}{BV_{0}+C_{T}D_{p}M\beta_{e}}\left({\dddot{x}}_{n}+\frac{1}{MV_{0}}f\right)\right|+\eta , \end{array}$$

where *K*_0_ = (*AD*_*p*_ *β*_*e*_/(*BV*_0_ + *C*_*T*_ *D*_*p*_ *Mβ*_*e*_))*K* and *D*_0_ = (*AD*_*p*_ *β*_*e*_/(*BV*_0_ + *C*_*T*_ *D*_*p*_ *Mβ*_*e*_))*D* are positive design parameters and *η* is sufficiently small positive parameter. On the assumption of (24), the robust control law is determined as

(25) $$\begin{array}{c} \omega_{p,\text{robust}}=D\zeta +K\mathrm{sgn}\left(\zeta \right). \end{array}$$

Finally, the sliding mode control law is selected as

(26) ωp=BV0+CTDpMβeADpβe×{x¨d+MV0BV0+CTDpMβex⃛m+(A2+BCTDp)βeBV0+CTDpMβex˙  +k1k3e˙+k2k3e}+Dζ+Ksgn(ζ).

If the total leakage coefficient *C*_*T*_ and the effective bulk modulus of EHA systems *β*_*e*_ are considered as variable parameters, that is, *C*_*T*_ = *C*_*T*,*n*_ + *C*_*T*,*p*_ and *β*_*e*_ = *β*_*e*,*n*_ + *β*_*e*,*p*_, then the uncertainty *ψ* is defined as

(27) ψ=θTφ=(MV0BV0+CT,pDpMβe,p)x⃛m+((A2+BCT,pDp)βe,pBV0+CT,pDpMβe,p)x˙,

where *θ* ^*T*^ = [(*MV*_0_/(*BV*_0_ + *C*_*T*,*p*_ *D*_*p*_ *Mβ*_*e*,*p*_)) ((*A* ^2^ + *BC*_*T*,*p*_ *D*_*p*_)*β*_*e*,*p*_/(*BV*_0_ + *C*_*T*,*p*_ *D*_*p*_ *Mβ*_*e*,*p*_))] and $\varphi =\left[\begin{array}{c} {\dddot{x}}_{m}\\ \dot{x} \end{array}\right]$. The parameter vector *θ* is considered as an unknown parameter vector, and it can be estimated by using the update law. From (26) and estimated unknown parameter vector $\hat{\theta }$, the estimated sliding mode control law can be selected as

(28) ω^p=BV0+CT,nDpMβe,nADpβe,n×{x¨d+θ^Tφ+MV0BV0+CT,nDpMβe,nx⃛m  +(A2+BCT,nDp)βe,nBV0+CT,nDpMβe,nx˙+k1k3e˙+k2k3e}+Dζ+Ksgn(ζ).

In order to obtain the update law for the unknown parameters, the Lyapunov candidate is defined as

(29) $$\begin{array}{c} V=\frac{1}{2}\zeta^{2}+\frac{1}{2\gamma }{\tilde{\theta }}^{T}\tilde{\theta }, \end{array}$$

where $\tilde{\theta }=\theta -\hat{\theta }$, *γ* is a positive parameter. Also, $\tilde{\theta }$ and $\hat{\theta }$ are the nominal and estimated parameter vectors, respectively. The derivative of the Lyapunov candidate including sliding dynamics is expressed as

(30) V˙=ζ[x¨d+θ^Tφ+MV0BV0+CT,nDpMβe,nx⃛m  +(A2+BCT,nDp)βe,nBV0+CT,nDpMβe,nx˙+k1k3e˙+k2k3e  −k3ADpβeBV0+CTDpMβeω^p]−1γθ~Tθ^˙.

Substituting the estimated angular velocity of the pump ${\hat{\omega }}_{p}$ given by (28) into (30) yields

(31) $$\begin{array}{c} \dot{V}=-D\zeta^{2}-K\zeta \mathrm{sgn}\left(\zeta \right)+{\tilde{\theta }}^{T}\left(\zeta \varphi -\frac{1}{\gamma }\dot{\hat{\theta }}\right). \end{array}$$

From the derivative of the Lyapunov included sliding dynamics, the update law for the unknown parameters is selected as

(32) $$\begin{array}{c} \dot{\hat{\theta }}=\gamma \zeta \varphi . \end{array}$$

Therefore, the derivative of the Lyapunov is given as

(33) $$\begin{array}{c} \dot{V}=-D\zeta^{2}-K\zeta \mathrm{sgn}\left(\zeta \right)\leq 0. \end{array}$$

By applying invariant set theorem for (33), the asymptotical stability of the EHA position control system is guaranteed [33, 34].

Finally, the sliding mode control law can be selected as

(34) ω^p=BV0+CTDpMβ^eADpβ^e×(θ^Tφ+MV0BV0+CT,nDpMβe,nx⃛m  +(A2+BCT,nDp)βe,nBV0+CT,nDpMβe,nx˙+k1k3e˙+k2k3e)+Dζ+Ksgn(ζ),

where ${\hat{\beta }}_{e}(t+T_{s})=\left(d{\hat{\beta }}_{e}(t)/dt\right)T_{s}+{\hat{\beta }}_{e}(t)$, ${\hat{C}}_{T}(t+T_{s})=\left(d{\hat{C}}_{T}(t)/dt\right)T_{s}+{\hat{C}}_{T}(t)$ and *T*_*s*_ is the sampling time.

## 5. Experimental Results and Discussion

This section looks to the experimental results in order to compare the performance of a position control systems. Three kinds of controllers are compared in order to show improved performance of overshoot reduction and robustness to the parameter change and load disturbance of the proposed controller for EHA position control system. 

**Figure 11:**
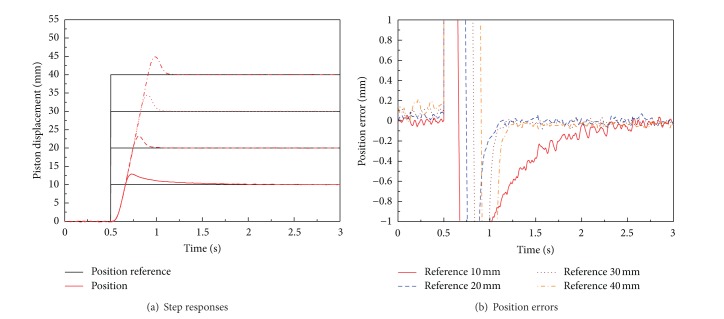
Performances of optimal PID position control system for EHA without load disturbance (only mass load is applied).

Firstly, optimal PID controller is designed and implemented by experiments.  Figure 11 shows experimental results of the PID position controller with step references of four different amplitudes which are 10, 20, 30, and 40 mm without load disturbance; that is, only mass load is applied as load to the position control system. 

The gains of the three-position controllers are optimally selected by using MATLAB/Simulink optimization toolbox based on identified system dynamic model of (14). In the process of optimal gain selection, the amplitude of position reference is set to 20 mm and target performance is 0.4 seconds of the rising time, 0.8 seconds of settling time, under 5% of percent overshoot, and under 0.1 mm of steady-state error. In experiments, the optimal gains derived from MATLAB/Simulink optimization toolbox are tuned to obtain best responses. The finally tuned optimal gains of the three controllers are represented in Table 3. 

**Figure 12:**
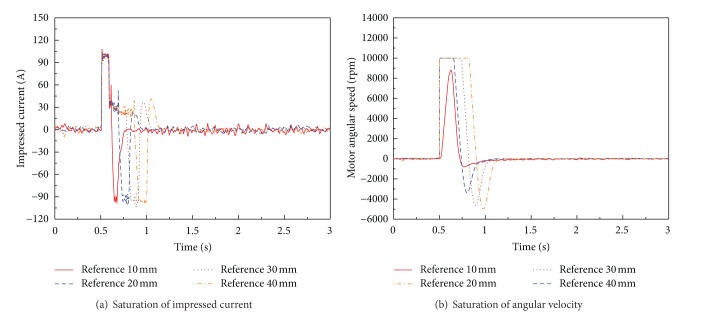
Saturation of BLDC motor for optimal PID position control system for EHA without load disturbance (only mass load is applied).

As shown in Figure 11, large overshoot is observed in position control result for using the optimal PID position controller. The performance of optimal PID position control system is represented in Table 4. The performance of optimal PID controller in experiment is not satisfactory the aimed value. In detail, large overshoot is observed in experiments. The current and angular velocity saturations of BLDC motor in position experiment result of optimal PID controller are shown in Figure 12. The large overshoot in position controller can be caused due to saturation of BLDC motor, as shown in Figure 12.

**Figure 13:**
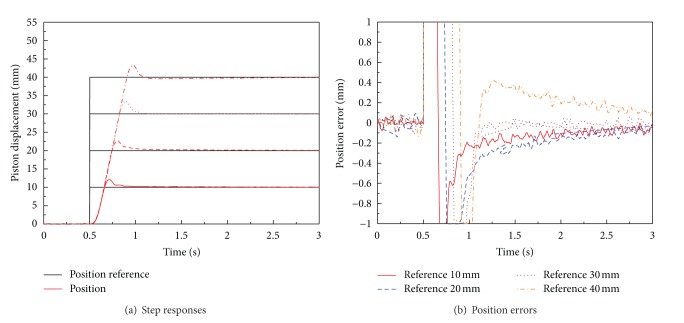
Performances of optimal antiwindup PID position control system for EHA without load disturbance (only mass load is applied).

To reduce such a large overshoot, an antiwindup algorithm which is described in Figure 9 is applied to the position control system of the EHA. The performance characteristics of an antiwindup PID position controller are depicted in Figure 13, and also the values of performance indices are shown in Table 4. As shown in Figure 13, the overshoot of the optimal antiwindup PID controller is more diminished than that of the optimal PID controller. However, as shown in Table 4, the antiwindup PID controller still has a performance of relatively long settling times which do not meet the aimed performance. Moreover, as the amplitude of the reference input increases, the steady-state error is increasing as shown in Figure 13.

**Figure 14:**
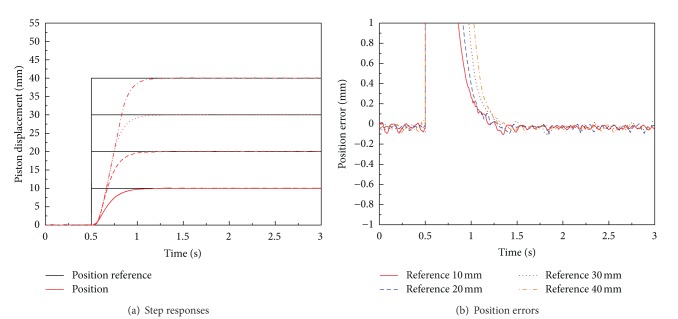
Performances of optimal adaptive antiwindup PID sliding mode position control system for EHA without load disturbance (only mass load is applied).

Figure 14 shows the experimental results of optimal adaptive antiwindup sliding mode position control system for the EHA system. As shown in Figure 14, the optimal antiwindup PID sliding mode position control system has a better performance than the system with optimal PID and antiwindup PID controllers such that relatively small overshoot and short settling time are obtained by the adaptive antiwindup PID sliding mode control system. As shown in Figure 14 and Table 4, the consistent performance is realized by the optimal adaptive antiwindup PID sliding mode control system. The time-domain quantitative performance indicators are also summarized in Table 4 for optimal adaptive antiwindup PID sliding mode control system without load.

**Figure 15:**
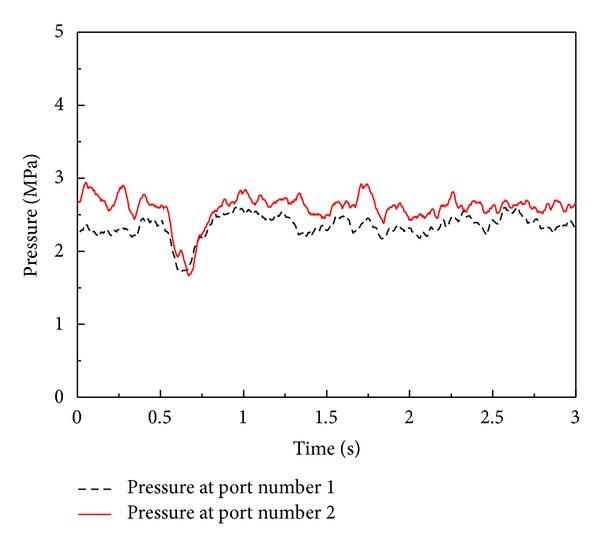
Pressure variations of optimal PID position control system for EHA without load disturbance (only mass load is applied).

**Figure 16:**
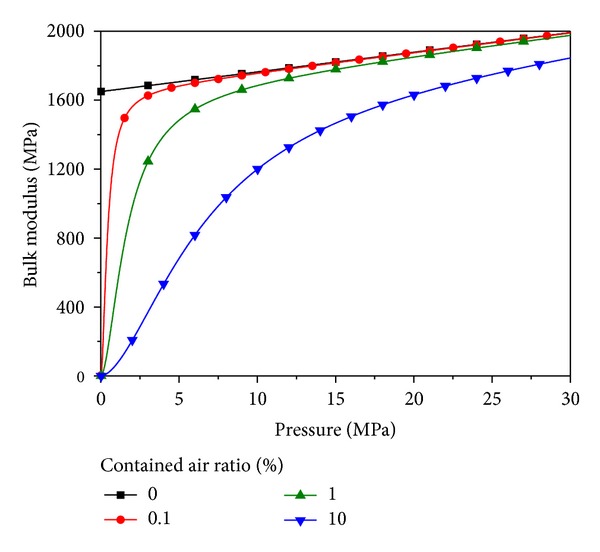
Example of the relationship between pressure and effective bulk modulus [12, 13].

On the other hand, Figure 15 depicts pressure variations of the optimal PID EHA position control system without load disturbance. As shown in Figure 15, the range of operating pressure of the EHA system is under 3 MPa in which the effective bulk modulus can be significantly changed according to contained air ratio of working fluid [5, 7, 11–13] as shown in Figure 16. The range of operating pressure for antiwindup PID and antiwindup PID sliding mode control system is also under 3 MPa.

**Figure 17:**
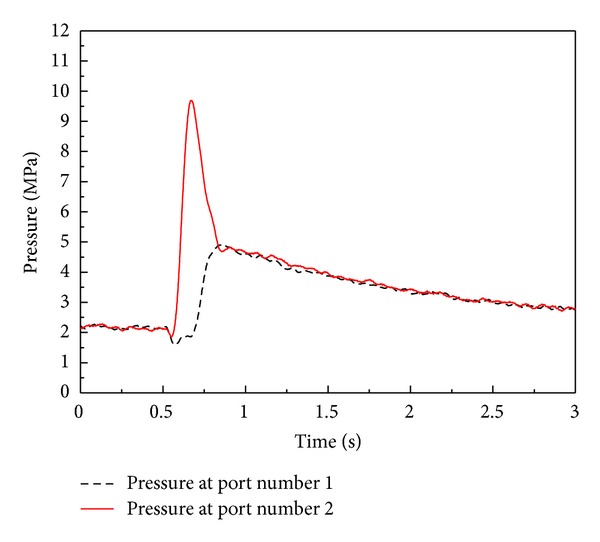
Pressure variations of optimal PID position control system for EHA with load disturbance.

Figure 17 depicts pressures of the EHA system when the position control systems are operating with load disturbance. As shown in Figure 17, the maximum operating pressures of the EHA system is about 10 MPa when the position control systems are working under the condition of adding disturbance load. The maximum operating pressure for antiwindup PID and antiwindup PID sliding mode control system is also up to 10 MPa. Thus, the effective bulk modulus can be changed greatly according to variation of operating pressure of the EHA systems. In addition, total leakage coefficient also can be changed in response to the level of working pressure.

In order to investigate robustness to the lumped system uncertainties such as varying parameters and modeling error and load disturbance, the performances of the three-position controllers of EHA system are compared in experiments by applying load disturbance. The experiments are conducted with 20 mm, position reference, and the load disturbance is applied by controlling the opening area of servo valve which is installed in a meter outline of load cylinder. 

**Figure 18:**
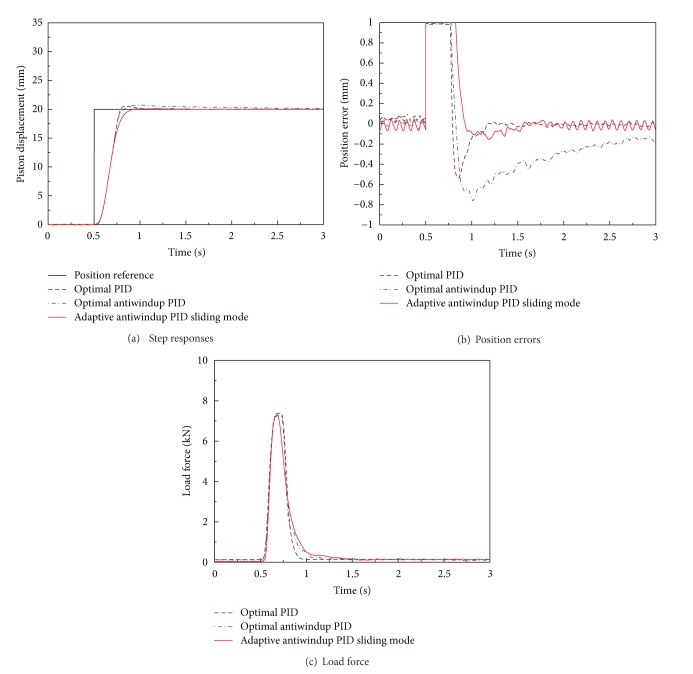
Performances of optimal PID, antiwindup PID, and adaptive antiwindup PID sliding mode control systems for EHA with load disturbance.

As shown in Figure 18, the optimal PID and optimal antiwindup PID control systems do not achieve robustness to the parameter change and load disturbance. In spite of the load disturbance and saturation of the BLDC motor in the position control system for EHA, the adaptive antiwindup PID sliding mode control system has a better performance and robustness than the optimal PID and optimal antiwindup PID control systems such that the adaptive antiwindup PID sliding mode control system recovers the parameter change and external disturbance in about 0.9 sec with a small overshoot. The time-domain quantitative performance indicators are summarized in Table 5 for optimal PID, antiwindup PID, and adaptive antiwindup PID sliding mode control systems with load disturbance. The list of terms are represented in Table 6.

## 6. Conclusion

This paper discussed the application of the optimal PID controller with optimal control gains using the optimization toolbox of MATLAB/Simulink to the position control system of EHA in order to understand the basic performance characteristics of the system. In experiment with optimal PID controller, large overshoot due to saturation of electric motor is observed. 

In order to achieve the desirable performance of EHA position control system, optimal antiwindup PID controller and adaptive antiwindup PID sliding mode controller are also applied to control the position of EHA systems. In the process of robust control system design, mathematical modeling was carried out based on theoretical analysis, and the simple third-order nominal model was derived. The system parameters of the simple second-order nominal model were identified by the signal compression method.

Based on the experimental results and the time-domain quantitative performance indicators, the performance of the EHA position control system can be improved and robust to the load disturbance, parameter variation, and system uncertainty such as saturation of electric motor by using the adaptive antiwindup PID sliding mode controller. 

## Figures and Tables

**Table 1:** Specification of components for the EHA prototype.

Specification	Value
Pump	
Type	Fixed displacement axial piston pump (bidirectional)
Number of pistons	7
Displacement	5 × 10^−6^ m^3^
Maximum pressure	20.6 × 10^6^ Pa
Motor	
Type	Brushless DC motor
DC link voltage	270 V
Rated current	52.4 Arms
Rated torque	13.4 N·m
Max speed	10,000 rpm
Power	9 kW
Hydraulic cylinder	
Type	Double rod, double acting
Piston diameter	0.108 m
Diameter of rod	0.044 m
Length of stroke	0.0650 m
Maximum pressure	20.6 × 10^6^ Pa
Accumulator	
Acc. Type	Bladder
Gas precharge pressure	0.49 × 10^6^ Pa
Accumulator volume	0.7 L
Load mass	
Weight	50 kg

**Table 2:** Specifications of the EHA prototype.

Specifications	Values
Stall force	Max. 1,541 N
Stroke	Max. ±33.7 mm
Velocity	123.0~142.5 mm/s
Power	Max. 9.0 kW

**Table 3:** Optimal gains of optimal PID, antiwindup PID, and adaptive anti-windup PID sliding mode control systems in experiments.

Gain	Optimal PID	Optimal antiwindup PID	Adaptive antiwindup PID sliding mode
Proportional (*K*_*p*_ or *k*_1_)	1034.3	1694.7	95.0
Integral (*K*_*i*_ or *k*_2_)	18.4	1087.7	0.001
Derivative (*K*_*d*_ or *k*_3_)	4.7	416.0	14.5
Antiwindup (*K*_*a*_)	—	0.001	0.001

**Table 4:** Comparison of the performance of optimal PID, optimal antiwindup PID, and adaptive antiwindup PID sliding mode control systems without load disturbance.

Performance	Reference	Optimal PID	Optimal antiwindup PID	Adaptive antiwindup PID sliding mode
Overshoot	10 mm	38.9%	20.8%	1.0%
	20 mm	16.1%	13.3%	0.5%
	30 mm	14.7%	11.9%	0.2%
	40 mm	12.2%	8.2%	0.1%

Settling time	10 mm	1.78 sec	2.2 sec	0.6 sec
	20 mm	0.56 sec	2.2 sec	0.6 sec
	30 mm	0.58 sec	0.63 sec	0.7 sec
	40 mm	0.69 sec	2.6 sec	0.7 sec

**Table 5:** Comparison of the performance of optimal PID, optimal antiwindup PID, and adaptive antiwindup PID sliding mode control systems with load disturbance.

Performance	Optimal PID	Optimal antiwindup PID	Adaptive antiwindup PID sliding mode
Overshoot	4.5%	3.9%	1.2%
Settling time	3.8 sec	1.8 sec	0.8 sec

**Table 6:** List of terms.

Notation	Description	Unit
*A *	Pressurized area of hydraulic cylinder of EHA	m^2^
*B*	Viscous friction coefficient	N/m/sec
*C*_*T*_	Equivalent leakage coefficient	m^3^/sec/Pa
*D*_*p*_	Pump displacement	m^3^/rad
*e*	Position error	mm
*i*	Current	Arms
*K*_pipe_	Pipe coefficient relating pressure drop to flow	Pa/m^6^/sec^2^
*K*_*s*_	Angular velocity to voltage gain	—
*K*_*p*_	Proportional control gain	—
*K*_*i*_	Integral control gain	—
*K*_*d*_	Differential control gain	—
*k*_*a*_	Antiwindup gain	—
*k*_1_, *k*_2_, *k*_3_,*D*, *K*, *γ*	Positive design parameters of adaptive PID sliding mode controller	—
*K*_*m*_, *T*_1_, *T*_*m*_1__, *T*_*m*_2__	Parameters of dynamic model for the BLDC motor with controller	—
*L *	Internal leakage coefficient of the cylinder	m^3^/Pa/sec
*M *	Mass load of position control test rig of EHA	N
*p*_1_, *p*_2_	Cylinder inlet/outlet chamber pressure	Pa
*p*_pipe_	Pressure drop of pipe between the pump and the cylinder	Pa
*Q*_1_, *Q*_2_	Cylinder inlet/outlet flow	m^3^/sec
*s*	Laplace operator	—
*T*_*m*_	Torque of BLDC motor	Nm/(rad/sec)
*T*_*s*_	Sampling time	sec
*V*_*a*_, *V*_*b*_	Volumes of inlet/outlet pipe and cylinder chamber	m^3^
*V*_0_	Mean volume of pipe and cylinder chamber.	m^3^
*v*_*e*_	Voltage signal of error for angular velocity of BLDC motor	V
*v*_*r*_	Voltage signal of reference angular velocity of BLDC motor	V
*v*_*ω*_	Voltage signal of angular velocity	V
*x*	Cylinder displacement	m
*y*_step_^*d*^(*t*)	Desired step response of the optimal PID and antiwindup PID control system	—
*y*_step_(*t*)	Step response by the identified transfer function	—
*β*_*e*_	Effective bulk modulus	Pa
ω_*p*_	Pump angular velocity	rad/sec
*ζ*	Sliding surface	—
ξ	Pump cross port leakage coefficient	m^3^/Pa/sec
